# The complete chloroplast genome sequence of *Ajania pacifica* (Nakai) Bremer & Humphries

**DOI:** 10.1080/23802359.2020.1750981

**Published:** 2020-06-12

**Authors:** Hyoung Tae Kim, Jung Sung Kim

**Affiliations:** a Institute of Agriculture Science and Technology, Chungbuk National University, Cheongju, Republic of Korea; b Department of Forest Science, Chungbuk National University, Cheongju, Republic of Korea

**Keywords:** *Ajania pacifica*, Chrysanthemum, Anthemideae, chloroplast genome

## Abstract

The complete chloroplast (cp) genome sequence of *Ajania pacifica,* called as golden and silver chrysanthemum, was newly analyzed in this study. It was 151,059 bp in length and was a typical circular structure composed of and comprised of a large single copy region (82,857 bp) and a small single copy region (18,294 bp) which were separated by two inverted repeat regions (24,954 bp). The molecular phylogenetic analyses of *A. pacifica* and its related taxa was conducted based on the complete chloroplast genome sequences, and it was proved that the genus *Ajania* is embedded in the genus *Chrysanthemum* clade as well as a monotypic genus *Opisthopappus*. In the other hand, the genus *Artemisia* was divided into two group in the tribe Anthemideae.

The Genus *Ajania* Poljakov is a member of daisy family. It belongs to the subfamily Asteroideae Lindley and the tribe Anthemideae Cass. within the family Asteraceae Berchtold & J. Presl (Oberprieler et al. 2007; Chase et al. 2016). The tribe Anthemideae is largely divided into two groups of *Artemisia-*group and *Chrysanthemum-*group even the debates on generic circumscription (Zhao et al. 2010; Liu et al. 2012), and *Ajania* is included in the latter group. Depend on the movement of the position, the genus *Ajania* had been included in the *Chrysanthemum sensu lato* or recognized a member of *Dendranthema* (DC.) Des Moul. before it was recently defined as an independent genera based on the feature of just disciform without ray floret. It is comprised of about 35–39 species and distributed in the temperate Asia, especially in China and Japan (Kadereit and Jeffrey 2007; *Ajania* at Flora of China website, http://www.efloras.org/florataxon.aspx?flora_id=2&taxon_id=100912). Among them, *A. pacifica* (Nakai) K. Bremer & Humphries is an endemic to Honshu of Japan and generally distributed along in the pacific coast (Ohashi and Yonekura 2004). But it has been introduced and cultivated in Korea recently.

We collected the plant material which was planted in the garden of Korea National Arboretum (37° 45.241′N, 127° 09.828′E, alt. 114 M) and the voucher (2019–1663) was deposited at the Herbarium of Chungbuk National University (CBNU). Complete chloroplast genome of *Ajania pacifica* (MN883841) was sequenced by HiSeq4000 of Illumina. Totally 31,846,800 paired-end reads (2 × 151bp) were obtained and 27,440,456 reads were used for the assemble to the reference sequence after trimming with the length range 50–151 bp. The assembled reads were *de novo* assembled using the Geneious assembler. Using the assembled contigs, we conducted to align and repeat the procedure up to make a single contig. Complete chloroplast genome was annotated using Geneious 10.2.6 (Kearse et al. 2012) with manual correction and tRNAScan-SE (Lowe and Eddy 1997) for tRNA gene. The average coverage of this chloroplast genome was 250.9. The phylogenetic tree was constructed with related Asteraceae members based on the concatenated 78 coding genes using RAxML (Stamatakis 2014).

The complete chloroplast genome of *Ajania pacifica* has a typical circular structure with 151,059 bp in length and comprised a large single copy region (LSC, 82,857 bp), a small single copy region (SSC, 18,294 bp), and two inverted repeat regions (IR, 24,954 bp). The GC contents was 37.5%. It was composed of 135 genes and they were identified 87 coding genes, 8 rRNA genes, and 37 tRNA genes. From the result of molecular phylogenetic analysis based on the complete chloroplast genome sequences, it was proved that the genus *Ajania* was embedded in the *Chrysanthemum* clade with strong support as well as monotypic genus *Opisthopappus* C. Shih (Figure 1). Besides, the genus *Artemisia* L. was apparently divided into two groups and *Crossostephium* Less. was a sister to one of them in the tribe Anthemideae. The relationship between *Ajania* and *Chrysanthemum* is one of the issue which has been studied by many researchers because of their distinguishable characteristics but confused phylogeny. The present data supported the closer relationship between them despite of morphological difference.

**Figure 1.**
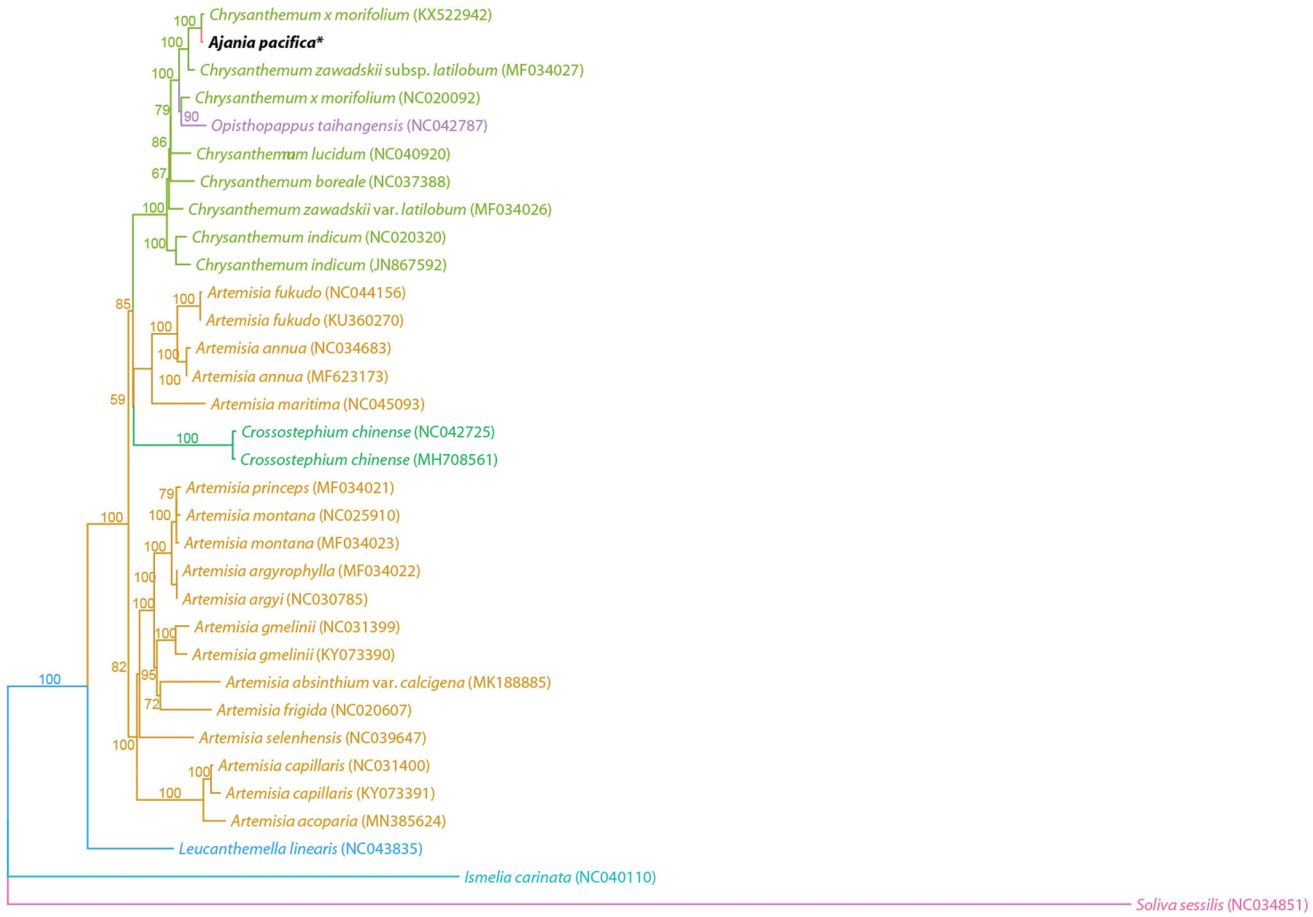
Phylogenetic tree of *Ajania pacifica* and related taxa using the complete chloroplast genome sequences.
